# Direct sac puncture versus transarterial embolization of type II endoleaks after endovascular abdominal aortic aneurysm repair: Comparison of outcomes

**DOI:** 10.1177/17085381231156661

**Published:** 2023-02-08

**Authors:** Bardia Moosavi, Youri Kaitoukov, Aline Khatchikian, Jason P Bayne, Andre Constantin, Errol Camlioglu

**Affiliations:** 1 Department of Radiology, 5620 McGill University, Montreal, QC, Canada; 2 Department of Radiology, 5620 McGill University Health Center, Montreal, QC, Canada; 3 Department of Vascular Surgery, Jewish General Hospital, Montreal, QC, Canada; 4 Department of Radiology, Jewish General Hospital, QC, Canada

**Keywords:** EVAR, Type 2 endoleak, direct sac puncture embolization

## Abstract

**Purpose:**

Type 2 endoleak (T2EL) is the most common type of endoleak after endovascular abdominal aortic aneurysm repair (EVAR), and increases the risk of aneurysm sac rupture if it persists beyond 6 months. The purpose of this study is to compare the efficacy and safety of direct sac puncture versus transarterial embolization of T2ELs.

**Methods:**

Retrospective review of 42 consecutive T2EL embolization procedures, 19 by DSP and 23 by transarterial technique, between January 2015 and December 2020. Primary outcome was aneurysm sac stability and resolution of endoleak at follow-up imaging. Adverse events (AE) were classified based on the Society of Interventional Radiology (SIR) practice guidelines.

**Results:**

Technical success was 94.7% (18/19) in the DSP group and 86.9% (20/23) in the transarterial group (*p* = 0.32 (−0.77–0.25)). Treatment efficacy was evaluated in 16 patients in the DSP group and 18 patients in the transarterial group who had follow-up imaging ≥6 months after embolization. Mean imaging follow-up was 17.1 ± 11.2 (range, 6–41) months in the DSP group and 26.5 ± 15.4 (range, 6–48) months in the transarterial group (*p* = 0.06, −19.24–0.37). Treatment efficacy was 75% (12/16) in the DSP group and 33.3% (6/18) in the transarterial group (*p* = 0.02, 95% CI, 0.09–0.97). There was no procedure-related mortality. Moderate-severe AE occurred in 15.7% (3/19) in the DSP group and 8.7% (2/23) in the transarterial group (*p* = 0.44, −0.12–0.26).

**Conclusion:**

In this study, DSP embolization of T2EL was equally safe and more effective than transarterial embolization in achieving aneurysm sac stability and resolution of endoleak.

## Introduction

Type 2 endoleak (T2EL) is the most common type of endoleak after endovascular abdominal aortic aneurysm repair (EVAR), occurring in up to 20% of cases.^1–3^ Approximately half of T2ELs resolve spontaneously.^1–3^ When T2EL persists beyond 6 months, it can lead to continued aneurysm sac growth and increased risk of rupture.^4^ Treatment options for persistent T2EL include transarterial embolization, direct sac puncture (DSP) embolization, endoscopic ligation of feeding arteries, and open repair.^5–10^

DSP embolization has shown technical and clinical success rates comparable to transarterial embolization in treating T2EL.^5^ Transarterial catheterization of T2EL after EVAR may be technically challenging, especially for iliolumbar or lumbar branches, and therefore transarterial embolization is typically limited to coils if the catheter cannot be advanced in the aneurysm sac.^11–13^ If the endoleak can be accessed percutaneously by DSP, embolization of both the endoleak nidus and inflow/outflow vessels can be achieved more readily, especially with liquid embolics.^14–19^

The purpose of this study is to compare the efficacy and safety of DSP versus transarterial embolization of T2ELs after EVAR.

## Methods

**Figure 1.**
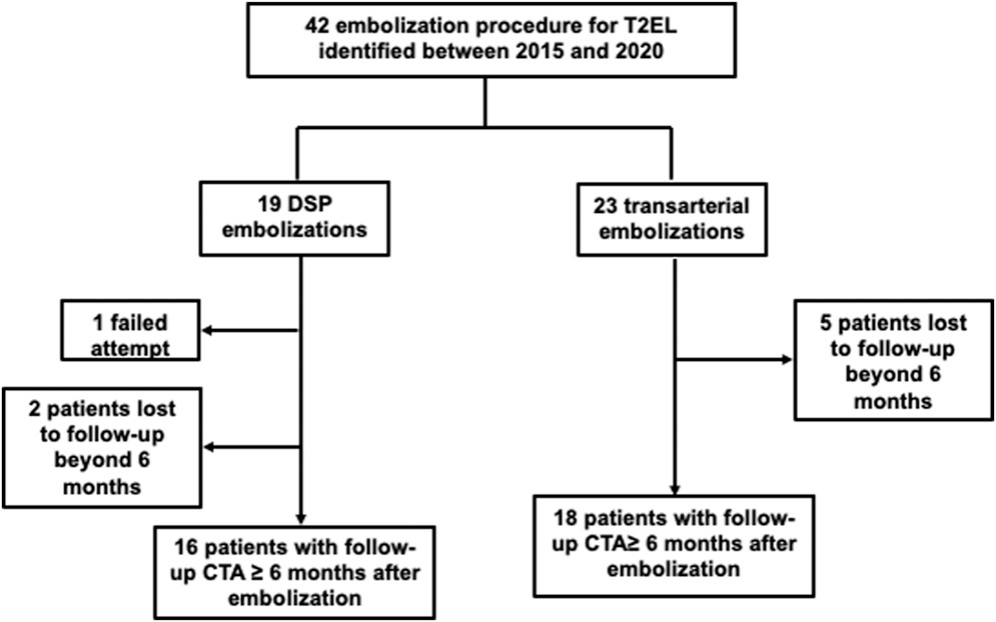
Study flow chart.

The study was authorized by our institutional research ethics board and need for informed consent was waived (protocol number 2021-2770). We retrospectively reviewed our imaging archiving system and identified 30 patients with T2EL after EVAR who underwent 42 consecutive embolization procedures at our tertiary care hospital between January 2015 and December 2020, 19 with direct sac puncture (DSP) and 23 with transarterial technique (Figure 1).

**Figure 3.**
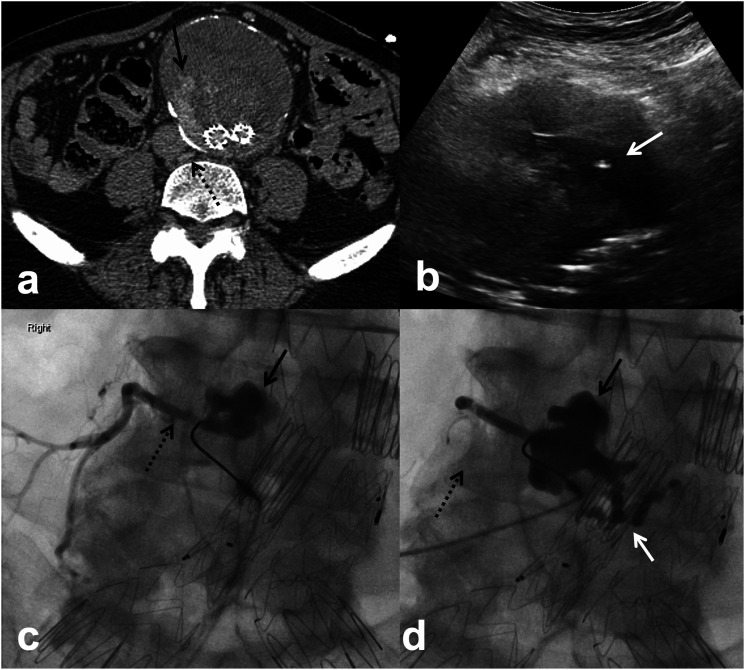
82-year-old man with previous EVAR in 2016 and transarterial coil embolization of the IMA for T2EL in 2017. Routine follow-up CTA in 2019 (a) demonstrated recurrent T2EL (solid arrow) which appeared to arise from a lumbar branch at L4 (dashed arrow). Direct sac puncture of the endoleak nidus was performed under US from a transabdominal approach (b) using a 22-G needle targeting the hypoechoic area within the sac (solid arrow). Initial angiography during contrast injection through the needle (c) demonstrates opacification of the endoleak nidus (solid arrow) and the right L4 lumbar artery (dashed arrow). The endoleak was subsequently embolized using 1:6 nBCA glue/Lipiodol mixture filling both the endoleak nidus (solid black arrow) and the right lumbar artery (dashed arrow). In addition, there was also filling of a left lumber artery (solid white arrow) not seen on initial angiography, suggesting that this may have been the inflow vessel. Follow-up CTA demonstrated aneurysm sac stability and resolution of endoleak at 2 years.

At our institution, post-EVAR imaging follow-up includes computed tomographic angiography (CTA) within 6 weeks, CTA at 6 and 12 months and annual CTA thereafter. Indication for embolization included persistent or recurrent T2EL and interval increase in aneurysm sac size by ≥ 0.5 cm. Embolizations were performed by one of four interventional radiologists with 4–30 years of experience. Choice of embolization technique was based on review of pre-procedure CTA. DSP was performed if a safe percutaneous window (transabdominal or translumbar) to the endoleak nidus was available and the feeding vessel of the endoleak was deemed difficult to catheterize endovascularly. For DSP, US-guided puncture of the endoleak nidus was performed with an 18 or 22-gauge needle from a transabdominal (*n* = 15) or translumbar (*n* = 4) approach. Once blood return observed, the needle was then connected to a syringe via a connecting tube and contrast was injected to demonstrate the endoleak nidus and the inflow and outflow vessels. Embolization was performed with n-butyl cyanoacrylate (nBCA) glue (Covidien, Duplin, Ireland) and ethiodized oil (Lipiodol, Guerbet, Villepinte, France) mixture in 16 of 18 (88.9%) of patients. In the remaining two patients, a combination of coils and glue (*n* = 1) or coils and gelatin sponge (Gelfoam, Pfizer, NY, USA; *n* = 1) were used. Ratio of nBCA glue to Lipiodol ranged between 1:1 and 1:6 which was determined by the interventional radiologist performing the procedure. Embolization with glue mixture was aimed at filling the inflow and outflow vessels in addition to filling the endoleak nidus whenever possible (Figure 3)

. In cases where coil embolization was performed, a 4-Fr catheter (DAV, Cook Medical, Bloomington, Indiana) or a 2.7-F microcatheter (Progreat, Terumo, Tokyo, Japan) was used. Transarterial embolization was performed via retrograde catheterization of the endoleak inflow vessel using a coaxial microcatheter system. The culprit vessel was determined based on pre-procedure review of CTA. After selective catheterization, the microcatheter was advanced as close as possible to the connection with the aneurysm sac. Embolization was performed with coils (*n* = 10), glue mixture (*n* = 7), coils and gelatin sponge (*n* = 3), coils and glue mixture (*n* = 2), or coils and thrombin (*n* = 1). Technical success was defined as complete endoleak embolization on intraprocedural fluoroscopy. Procedure-related complications were classified according to the new Society of Interventional Radiology (SIR) classification of adverse events (AE).^20^

**Figure 2.**
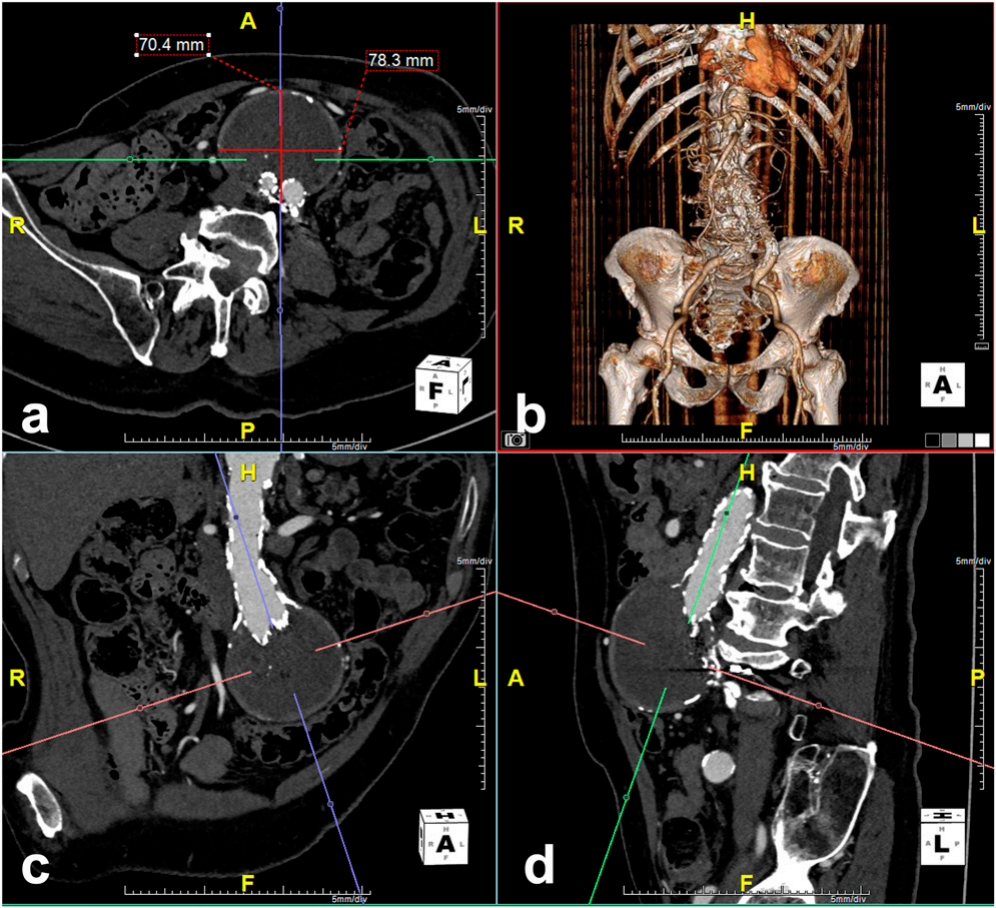
77-year-old man with previous EVAR in 2014. Follow-up CTA in 2016 demonstrated T2EL from a lumbar branch (not shown) and increased aneurysm sac diameter by 11 mm. Aneurysm sac measurements were performed using a three-dimensional post-processing software (TeraRecon, Foster City, California). Anteroposterior (AP) and transverse (TR) measurements of the aneurysm sac (measurements in a) were obtained in the true axial plane (red line in c and d) after adjustments made in the sagittal (blue line in a and b) and coronal (green line in a and c) planes to correct for the tortuosity and angulation of the aorta.

Clinical success was defined as aneurysm sac stability and resolution of T2EL on follow-up imaging. Treatment efficacy was evaluated in 16 patients in the DSP group and 18 patients in the transarterial group who had follow-up CTA after embolization (Figure 1). All CTA images were reviewed by a fellowship-trained interventional radiologist. Aortic measurements were performed on a three-dimensional post-processing software (TeraRecon, Foster City, California) allowing measurement of the largest aortic dimensions in the true axial plane (Figure 2).

Proportions were compared using t-test of proportions, continuous variables were compared with Student’s t-test, and categorical variables were compared with Fisher’s exact test. p values less than 0.05 were considered statistically significant. Statistical analysis was performed with Stata software (StataCorp, College Station, Texas). 

## Results

Patient demographics and pre-procedural mean aneurysm sac diameter are summarized in Table 1 and procedural information and outcomes are summarized in Table 2.

**Table 1.** Patient demographics and pre-procedure mean sac diameter.

	Direct sac puncture (*n* = 19)	Transarterial (*n* = 23)	*p*-value (95% CI)
Mean age	81.3 ± 5.4 (range, 68–92)	78.8 ± 6.9 (range, 68–92)	0.19 (−1.39–6.48)
Gender			
Male	16	20	1.0
Female	3	3	
Mean aneurysm sac diameter at index treatment	6.5 ± 1.5 cm (range, 5.2–11.5)	6.1 ± 1.0 cm (range, 5.2–9.5)	0.08 (−0.08–1.54)

**Table 2.** Procedural parameters and outcomes.

	Direct sac puncture (*n* = 19)	Transarterial (*n* = 23)	*p*-value (95% CI)
Technical success	94.7% (18/19)	86.9% (20/23)	0.32 (−0.77–0.25)
Culprit vessel			
Inferior mesenteric artery	2	5	--
Lumbar artery	13	8	--
Iliolumbar artery	1	9	--
Median sacral artery	--	1	--
inferior Gluteal artery	2	--	--
Material used			
Glue	88.9% (16/18)	30.4% (7/23)	--
Coils	0%	43.5% (10/23)	--
Coils and glue	5.5% (1/18)	8.7% (2/23)	--
Coils and gelfoam	5.5% (1/18)	13% (3/23)	--
Coils and thrombin	0%	4.3% (1/23)	--
Mean follow-up imaging (mean±SD, range)	17.1 ± 12.3 (range, 6–41) months	26.5 ± 15.4 (range, 6–48) months	0.06 (−19.24–0.37)
Aneurysm sac stability	75% (12/16)	33.3% (6/18)	0.02 (0.09–0.97)
Mean increase in aneurysm sac diameter from index embolization	0.13 ± 0.8 (range, −1.1–2.4)	0.49 ± 0.5 (range, −0.4–1.4)	0.16 (−0.79–0.14)
Adverse events			
Moderate	2	1	0.44 (−0.12–0.26)
Severe	1	1	

All patients had undergone endovascular repair of an abdominal aortic (*n* = 25) or aortoiliac (*n* = 5) aneurysm with a either a Zenith Flex endovascular stent graft (Cook Medical, Bloomington, Indiana) or Endurant II stent graft (Medtronic, Dublin, Ireland). The median interval between EVAR and first embolization was 25.5 months (range, 2–78 months). Technical success was 94.7% in the DSP group and 86.9% in the transarterial group (95% CI -0.77–0.25, *p* = 0.32). The endoleak nidus could not be accessed percutaneously in one patient in the DSP group and therefore embolization was not performed. In the transarterial group, the inflow artery from an iliolumbar branch could not be catheterized in one patient. Another patient underwent two transarterial embolizations within 5 days which failed to adequately control the endoleak due to the complex nature of the endoleak in a fenestrated stent graft; the patient underwent graft revision 3 days following the second failed embolization.

Mean sac diameter before embolization was 6.5 ± 1.5 cm (range, 5.2–11.5) in the DSP group and 6.1 ± 1.0 cm (range, 5.2–9.5) in the transarterial group (95% CI, −0.08–1.54, *p* = 0.08). Mean imaging follow-up was 17.1 ± 12.3 (range, 6–41) months in the DSP group and 26.5 ± 15.4 (range, 6–48) months in the transarterial group (95% CI -19.24–0.37, *p* = 0.06). There was aneurysm sac stability and resolution of endoleak in 12 of 16 (75%) patients in the DSP group and 6 of 18 (33.3%) patients in the transarterial group (95% CI, 0.09–0.97, *p* = 0.02).

There was recurrent endoleak in four patients in the DSP group. One patient who had endoleak recurrence and sac growth 8 months after DSP embolization underwent open graft repair. One patient had recurrent endoleak at 6 months with 0.3 cm increase in mean sac diameter and underwent transarterial embolization of the median sacral artery, but there was continued gradual increase in mean sac dimeter by 0.8 cm over 41 months of imaging follow-up. No further intervention was performed in two patients with <0.5 cm annual sac growth over 2 years and 3 years, respectively.

In the transarterial group, 12 patients had endoleak recurrence. Six patients underwent repeat embolization with DSP technique 22.5 ± 16 months (range, 3–40) after the index embolization achieving endoleak control and sac stability in 83.3% (5/6) over 14 ± 12.1 months (range, 6–34 months) of further imaging follow-up; one patient had continued sac growth by 0.5 cm at 9 months and underwent graft revision. Two patients underwent repeat embolization with transarterial technique 19 ± 4.2 months (range, 16–22) after the index transarterial embolization; both patients developed recurrent T2EL and required a third embolization with DSP technique at 28 ± 21.2 months (range, 13–43) with no further increase in mean sac diameter after an additional 12 ± 7.1 months (range, 7–17) of imaging follow-up. The graft was surgically revised in one patient at 9 months. No intervention was performed in one patient with <0.5 cm annual sac growth over 3 years. One patient with endoleak recurrence at 13 months was lost to further follow-up. Another patient with endoleak recurrence and gradual sac growth underwent failed attempt at repeat embolization at 26 months and was lost to further follow-up.

There was no procedure-related mortality. Procedure-related complications occurred in 3 of 19 (15.7%) patients in the DSP group and 2 of 23 (8.7%) patients in the transarterial group (95% CI -0.12–0.26, *p* = 0.44). In the DSP group, severe AE occurred in one patient who developed low back and thigh pain during post-procedural observation prompting a CT scan which demonstrated non-target embolization of glue mixture in the right inferior gluteal artery. The patient was admitted overnight for pain management and recovered without further clinical consequence. Moderate AE occurred in two patients in whom there was glue mixture extravasation during embolization. Both patients developed severe abdominal pain, but remained hemodynamically stable. CT confirmed glue mixture outside of the aneurysm sac but no evidence of active bleeding. Both patients were successfully managed with analgesics and prolonged outpatient observation. In the transarterial group, severe AE occurred in one patient where non-target embolization of glue mixture in the superior rectal artery resulted in one episode of rectal bleeding in the recovery room. The patient was admitted for overnight observation. Moderate AE occurred in another patient who presented to the hospital 3 days after the procedure complaining of anterolateral thigh pain, presumed to be from non-target embolization of glue mixture. This patient was treated with outpatient oral anti-inflammatory medication.

DSP embolization was performed with glue mixture in 16 of 18 (88.9%) patients, two of whom were lost to follow-up. There was freedom from sac growth in 11 of 14 (78.6%) patients at 16.5 ± 12.1 months (range, 6–41) post embolization. Embolic filling of inflow/outflow vessels in addition to the aneurysm sac was observed in 9 of 18 (50%) patients and filling of the sac alone was demonstrated in the remaining 9 patients. There was aneurysm sac stability in all patients in whom both the aneurysm sac and inflow/outflow vessels were embolized. In 9 patients in whom aneurysm sac only was embolized, four had recurrent endoleak on follow-up imaging as discussed above.

## Discussion

T2EL is the most common complication following EVAR, occurring in approximately 1 in 5 cases.^1–3^ Most early T2ELs resolve spontaneously^1–3^ but up to 20% persist and may lead to aneurysm sac growth with increased risk of rupture.^4^ The society of vascular surgery (SVS) practice guidelines recommends intervention when T2EL persists beyond 6 months and there is an increase in aneurysm size of >0.5 cm.^21^ Previous studies have reported high technical and clinical success of DSP embolization in treating T2EL.^5,6,14,15^ In this cohort, DSP embolization achieved a significantly higher clinical efficacy compared with transarterial embolization (75% vs 33.3%).

Previous studies have reported similar treatment outcomes of DSP versus transarterial embolization, and there has been increasing support for DSP approach to embolization of T2EL over the past decade.^6,11,17,19^ Yang et al.^6^ have reported 64% treatment efficacy with DSP and 57% with transarterial embolization in a mean follow-up period of 21.8 months.^6^ Stavropoulos et al. have reported 72% clinical success with DSP embolization and 78% with transarterial embolization in a mean follow-up period of 18.7 months.^19^ Similarly, Massis et al. have reported DPS and transarterial embolization success rates at 75% and 72.4%, respectively, although within a substantially shorter mean follow-up period of 3.8 months.^17^ Treatment efficacy of DSP embolization in our study at 75% compares favorably to previous reports. Treatment efficacy of transarterial embolization in our study at 33.3% was lower compared to that reported by Yang et al.^6^ within a similar follow-up period although a sizeable proportion (about 40%) of patients in their study had more than one embolization procedure.^6^ Treatment efficacy of transarterial embolization in our study was also lower than that reported by Stavropoulos et al. and Massis et al., but this discrepancy may be partly due to longer follow-up period of 26.5 months in our study compared to 18.7 months and 3.8 months in the two prior studies, respectively.^17,19^ Transarterial embolization results in our study are comparable to that by Abularrage et al. who reported 40% success rate in a follow-up period of 13.7 months.^11^

The rate of T2EL recurrence in our study is similar to previous studies where endoleak recurrence has been reported to be between 20 and 30% after DSP embolization and 20–70% after transarterial embolization.^6,12,17,19^ In this study, there was endoleak recurrence in four patients (25%) and 12 patients (66.6%) after DSP embolization and transarterial embolization, respectively. The wide range of endoleak recurrence rate after transarterial embolization reported in the literature may be partly related to technique and variability in embolization material used. As in previous reports, endoleak recurrence following transarterial embolization in our study was mainly from recruitment of new arterial branches and not the previously embolized artery.^6,11,13^

A variety of embolization material has been described for T2EL embolization. Decision on type of embolic material mainly depends on the anatomy of the endoleak.^22^ Transarterial catheterization of inflow/outflow vessels may be technically challenging, especially for iliolumbar or lumbar branches, and therefore transarterial embolization is typically limited to coils if the catheter cannot be advanced in the aneurysm sac.^11–13^ On the other hand, if the endoleak nidus can be accessed percutaneously, embolization of both the nidus and inflow/outflow vessels can be achieved more readily with DSP technique, which is reflected in higher technical success rates of DSP versus transarterial embolization.^5^ The use of liquid embolics in DSP embolization is favorable since selective embolization of inflow/outflow vessels may be challenging or not possible to perform depending on the orientation of the vessel in relation to the percutaneous access site. Prior studies describing the use of liquid embolics in DSP embolization have shown high clinical success rates,^11,17,18,23^ but few have reported on using glue mixture alone.^6,14^ In this study, embolization was performed with glue mixture in the majority (88.9% [16/18]) of cases, achieving 78.6% clinical success. Our results are similar to those reported by Zener et al. who used glue mixture in 15 patients and achieved 75% clinical success within a comparable follow-up period (16.5 months in this study vs 15.5 months in the study by Zener et al.). Yang et al. report on using glue mixture in approximately 17 DSP embolizations achieving 91% clinical success rate at 24 months although a third of those cases had more than one embolization, likely explaining the higher success rate in their study compared to our study. Transcaval embolization of T2EL is an alternative technique for T2EL embolization which has shown high technical success rate and safety profile although it is not commonly performed and the quality of the available data is limited.^24^ Transcaval embolization was not performed in our series.

In this study, DSP embolization of inflow/outflow vessels in addition to the aneurysm sac resulted in higher clinical success rate compared to embolization of the sac alone. Embolization of the inflow/outflow vessels was not demonstrated in all four patients who failed DSP embolization. This suggests that embolization of the inflow/outflow vessels in addition to the endoleak nidus is more likely to prevent new channels from forming within the aneurysm sac. This may also explain the higher clinical success rates seen with DSP embolization with glue mixture compared to coils since coil embolization of the aneurysm sac alone does not necessarily achieve inflow/outflow vessel occlusion.^13,14,18^ Similar clinical success rates of DSP with Onyx supports this observation.^11, 17^ Abularrage et al. reported 76% clinical success rate when using Onyx compare to 36% with coil embolization alone.^11^

Procedure-related complications occurred in 15.7% (3/19) in the DSP group which is higher than the 0–12% range reported in previous studies.^6,14,16,25^ In the DSP group, one severe AE occurred as a result of non-target embolization of glue mixture, the incidence of which was similar to previous studies describing DSP embolization technique.^6,14,19^ While there may be concern for non-target embolization of glue by attempting retrograde embolization of the inflow/outflow vessels via DSP as in this study, non-target embolization can be avoided by adjusting the nBCA glue to Lipiodol ratio based on the rate of filling of the vessels during initial angiography. The two other AEs in this study were glue extravasation outside of the aneurysm sac resulting in abdominal pain. These AEs were considered moderate and only required prolonged observation with no further clinical consequence. Glue extravasation was not been reported in the study by Zener et al. despite having performed a higher number if transabdominal punctures (33 vs 15 in this study). Puncture site hematoma did not occur in our study but has been described with DSP technique including formation of rectus sheath hematomas.^6,14^ Procedure-related complications occurred in 8.7% (2/23) in the transarterial group in this study, both of which were result of non-target embolization. While this is an uncommon complication of transarterial embolization of T2ELs, the higher rate in our study may be explained by insufficient proximity of the microcatheter to the aneurysm sac during embolization and more frequent utilization of liquid embolics compared to previous reports.^17,19^

Our study has some limitations. First, the study was performed retrospectively with a relatively small sample size. Second, the mean follow-up period after DSP embolization was shorter than that available for transarterial embolization which limits comparison of durability between the two techniques. In our study, freedom from aneurysm sac growth after DSP embolization decreased from 81.3% at 16.1 months to 75% at 24.3 months in 50% of patients who had follow-up imaging ≥12 months after embolization, which is still higher than the 44.4% clinical success rate of transarterial embolization within a comparable follow-up period. Finally, aneurysm sac volumes were not measured in this study. Aneurysm sac growth was determined by increase in the largest mean sac diameter measured at the same level on both pre-procedure and follow-up imaging. While this method is less accurate than calculating the change in aneurysm sac volume, it is more reflective of clinical practice.

In conclusion, DSP embolization of T2EL after EVAR was equally safe and more effective than transarterial embolization in achieving freedom from aneurysm sac growth and resulted in lower rate of endoleak recurrence. Larger studies with longer follow-up periods are needed to confirm these findings.
